# The effects of flip angle and gadolinium contrast agent on single breath-hold compressed sensing cardiac magnetic resonance cine for biventricular global strain assessment

**DOI:** 10.3389/fcvm.2024.1286271

**Published:** 2024-01-29

**Authors:** Fuyan Wang, Cailing Pu, Siying Ma, Junjie Zhou, Yangyang Jiang, Feidan Yu, Shuheng Zhang, Yan Wu, Lingjie Zhang, Chengbin He, Hongjie Hu

**Affiliations:** ^1^ Department of Radiology, Sir Run Run Shaw Hospital, Zhejiang University School of Medicine, Hangzhou, Zhejiang, China; ^2^ United Imaging Healthcare, Shanghai, China

**Keywords:** compressed sensing, cardiovascular magnetic resonance, cine, strain, feature tracking

## Abstract

**Background:**

Due to its potential to significantly reduce scanning time while delivering accurate results for cardiac volume function, compressed sensing (CS) has gained traction in cardiovascular magnetic resonance (CMR) cine. However, further investigation is necessary to explore its feasibility and impact on myocardial strain results.

**Materials and methods:**

A total of 102 participants [75 men, 46.5 ± 17.1 (SD) years] were included in this study. Each patient underwent four consecutive cine sequences with the same slice localization, including the reference multi-breath-hold balanced steady-state free precession (bSSFP_ref_) cine, the CS cine with the same flip angle as bSSFP_ref_ before (CS_45_) and after (eCS_45_) contrast enhancement, and the CS cine (eCS_70)_ with a 70-degree flip angle after contrast enhancement. Biventricular strain parameters were derived from cine images. Two-tailed paired t-tests were used for data analysis.

**Results:**

Global radial strain (GRS), global circumferential strain (GCS), and global longitudinal strain (GLS) were observed to be significantly lower in comparison to those obtained from bSSFP_ref_ sequences for both the right and left ventricles (all *p* < 0.001). No significant difference was observed on biventricular GRS-LAX (long-axis) and GLS values derived from enhanced and unenhanced CS cine sequences with the same flip angle, but remarkable reductions were noted in GRS-SAX (short-axis) and GCS values (*p* < 0.001). After contrast injection, a larger flip angle caused a significant elevation in left ventricular strain results (*p* < 0.001) but did not affect the right ventricle. The increase in flip angle appeared to compensate for contrast agent affection on left ventricular GRS-SAX, GCS values, and right ventricular GRS-LAX, GLS values.

**Conclusion:**

Despite incorporating gadolinium contrast agents and applying larger flip angles, single breath-hold CS cine sequences consistently yielded diminished strain values for both ventricles when compared with conventional cine sequences. Prior to employing this single breath-hold CS cine sequence to refine the clinical CMR examination procedure, it is crucial to consider its impact on myocardial strain results.

## Introduction

CMR is a noninvasive, multi-parametric modality that has firmly established itself as a valuable diagnostic tool across a range of clinical scenarios, encompassing both ischemic and non-ischemic heart diseases (1). Myocardial strain, as a metric indicating myocardial deformation across the cardiac cycle, holds the ability to reveal abnormalities in the initial phases of various cardiovascular diseases (CVDs) (2). MRI strain techniques, such as strain-encoded imaging (SENC), myocardial tagging, displacement-encoding with stimulated echoes (DENSE), and tissue phase mapping, are hindered by their limited availability (3). In recent years, CMR has swiftly emerged as a compelling alternative to speckle tracking echocardiography, with this transition largely driven by the innovative feature-tracking (FT) technique. This technique evaluates strain by analyzing standard long-axis and short-axis view cine images (4, 5). The CMR-FT method has undergone thorough exploration utilizing sets of readily obtainable 2D cine images, characterized by a standard slice thickness of 6–8 mm and a clear demarcation between the blood pool and myocardium (6). Harnessing CMR-FT for myocardial strain analysis holds great potential for improving the detection of myocardial dysfunction, and then offering additional diagnostic and prognostic insights (7–9).

Compressed sensing (CS) represents a rapidly advancing magnetic resonance technology that offers a vital approach to reducing MR acquisition time (10). This is accomplished through the utilization of significantly undersampled k-space data and rapid iterative reconstruction techniques (11). CS technique has successfully shortened cine imaging in half the time compared to conventional cine sequences, while still maintaining comparable volumetric values (12). A series of previous studies have consistently validated its effectiveness in evaluating cardiac volume among patients with various kinds of CVDs (13–15). Despite the rapid and widespread adoption of CS technology in CMR cine, a lack of established standards for image acquisition and interpretation, as well as variability introduced by various software vendors, necessitates further research to comprehensively understand the differences in strain values between conventional cine and CS-based cine (7).

The main objective of this study is to evaluate the influence of gadolinium contrast agents, flip angle, and retrospective ECG gating CS-based sampling and reconstruction methods, on strain parameters derived from cine-based CMR-FT during clinical CMR examinations. This single breath-hold CMR CS-based bSSFP cine (called CS cine hereafter) sequence combines CS and SENSE (sensitivity encoding) as an acceleration technique, known as uCS 2.0 (detailed in methods part) by United Imaging Healthcare, which allows for the acquisition of 8–12 short-axis ventricular slices in a single breath-hold using retrospective ECG gating. This approach maintains identical spatial resolutions and a slightly longer temporal resolution to those of the standard bSSFP cine sequence used as a reference.

## Materials and methods

### Study population

**Figure 1:**
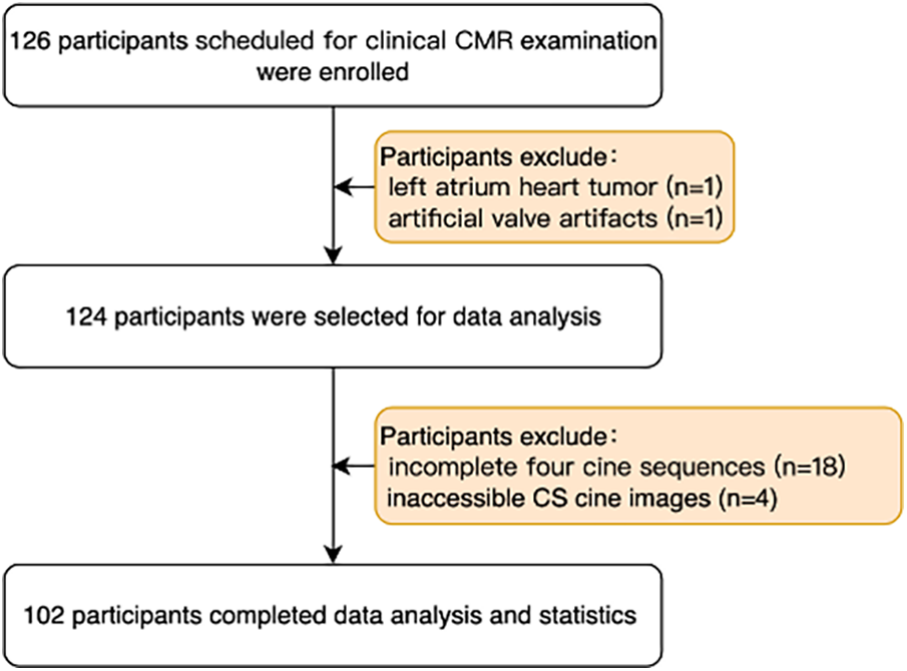
Enrollment flowchart of the participants. CS, compressed sensing.

Between January 2022 and August 2022, we prospectively enrolled 126 patients routinely scheduled for clinical CMR in this study. Prior to the CMR examination, all patients underwent a pre-evaluation to ensure their renal function (eGFR: >40 ml/min), heart rate (less than 90 bpm), and breath-hold duration (more than 15 s) were suitable for the test. Following the examination, the quality and artifacts of cine images were manually checked. Exclusions included two patients with left atrium heart tumor and artificial valve artifacts separately, two patients with left atrium heart tumor and artificial valve artifacts separately, 16 patients (15 patients who could not finish all short-axis bSSFP_ref_ cine segments scanning due to poor breath-hold ability or unpredictable arrhythmia, one CS cine of a patient was skipped by misoperation of the technician) with incomplete four comparable cine series, and four patients (BMI all higher than 30 kg/m^2^, could not discern the heart contour from the reconstructed cine images) with inaccessible CS cine images were excluded. Ultimately, 102 patients were included in the final analysis (inclusion details depicted in Figure 1). This study was conducted in accordance with the Declaration of Helsinki and received approval from the Ethics Committee of Sir Run Run Shaw Hospital (approval no. 2022-0212). Informed consent was obtained from all participants or their surrogates.

### Data acquisition

**Figure 2:**
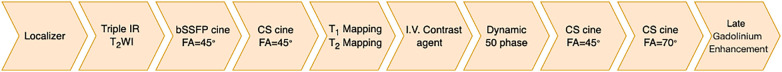
CMR scanning procedure. CS, compressed sensing; bSSFP, balanced free steady state precession.

All CMR examinations were conducted on a 1.5 T MRI scanner (uMR 680, United Imaging Healthcare, Shanghai, China) equipped with a 24-channel dedicated cardiac coil. Four standard views of the heart (two chamber, four chamber, three chamber, and short-axis) were captured referring to either automatically reconstructed 3D scout or manual localization images. The short-axis cine consisted of 8–12 slices, aligned with the mitral valve and covering the entire left ventricle (LV). For CS cine, a 2D balanced steady-state sequence was modified to achieve high spatial and temporal resolution imaging by utilizing highly under-sampled data acquisition. The sampling pattern in k-space follows the VALAS (Variable spatial-temporal Latin hypercube and echo-Sharing) principle, which is a design approach that generates a constrained distribution of sample positions on a spatial-temporal grid with reduced statistical fluctuation (16). Each cardiac phase consists of 15 phase encoding lines, and each 2D slice is acquired over two heartbeats. The reconstruction process involves using compressed sensing (CS) and utilizes spatial and temporal sparsity regularization in the total variation domain. To optimize the cost function, which includes data fidelity and total variation terms, an iterative reconstruction method with 80 iterations was employed (17). Every patient underwent four times of standard view (2 chamber, 3 chamber, 4 chamber and short axis) cine sequence scanning: first, the reference retrospective ECG-gated conventional multi breath-hold segmented bSSFP cine sequence (bSSFP_ref_) with a 45° flip angle was scanned, then the CS-based single breath bSSFP cine sequence with a 45° flip angle was acquired (CS_45_), after enhancement agent injection (Gadodiamide, 3.0 ml/s, 0.15 mmol/kg), the CS cine sequence with the same parameters as CS45 (eCS_45_) and the CS cine only with a larger flip angle (70°, eCS_70_) was acquired, the detailed scanning procedure and imaging parameters were concluded in Figure 2 and Table 1.

**Table 1:** Imaging parameter of the reference balanced steady-state free precession cine and compressed sensing cine with different flip angles.

Parameter	bSSFP_ref_	CS_45_	CS_70_
Sequence	2D bSSFP cine	2D bSSFP cine	2D bSSFP cine
ECG mode	Retrospective	Retrospective	Retrospective
Spatial resolution (mm^2^)	1.88 × 1.88	1.88 × 1.88	1.88 × 1.88
Field of view (mm^2^)	360 × 320	360 × 320	360 × 320
Matrix	192 × 171	192 × 171	192 × 171
Slice thickness (mm)	8	8	8
Repetition time (ms)	3.12	2.86	2.86
Echo time (ms)	1.51	1.34	1.34
Flip angle(degrees)	45	45	70
Temporal resolution (ms)	31.2	42.9	42.9
Bandwidth (Hz/pixel)	1,200	1,200	1,200
Cardiac phase (n)	25	25	25
Acceleration factor	2	11.4	11.4
Number of breath-hold (short-axis, n)	9.1 ± 0.6	1	1
Number of iterative reconstruction (n)	–	80	80

bSSFP, balanced free steady state precession; CS, compressed sensing.

### Data analysis

All eligible cine CMR images were analyzed on strain module of commercially available CVI^42^ software (Circle Cardiovascular Imaging, version 5.13.5, Calgary, Alberta, Canada) by one experienced radiologist (FYW, 7 years of CMR experience). The analysis of LV and RV strain was performed using feature tracking based on optical flow technology (18, 19). Biventricular endocardial and epicardial contours were automatically drawn and manually verified on end-diastolic phase cine images for all series concurrently. During strain analysis, papillary muscles were excluded. The peak systolic global longitudinal strain (GLS) value was derived from two-, three-, and four-chamber long-axis views. The peak systolic global circumferential strain (GCS) was derived from a basal, mid, and apical slice in the short-axis view. The peak systolic global radial strain (GRS) value was derived from cine images in both short-axis and long-axis views, with the average of basal, midventricular, and apical short-axis sections taken into account. To evaluate the reproducibility of LV strain values, two independent observers with 3 and 5 years of CMR experience (YW and CLP, respectively) were selected to assess 20 randomly chosen subjects in a blinded manner. For assessing the intra-observer reproducibility (by YW), a six-week interval was chosen between the first and second analyses. Image contrast was quantitatively characterized by the blood pool-to-myocardial signal intensity ratio. In all patients, blood pool and myocardial signal intensity values were assessed in the end-diastolic midventricular short-axis cine image. The myocardial signal intensity represented the mean pixel intensity within a circular region of interest positioned in the middle septum. This region of interest had a diameter approximately two-thirds the width of the septum. The blood pool signal was determined by employing a same-sized region of interest across four cine series located in the center of the left ventricular cavity. The formula used was: SI_pool−to−myo_ = SI_pool/SImyo_ (SI, signal intensity) (15).

### Statistical analysis

Statistical analysis was conducted using IBM SPSS (v. 26.0) and GraphPad Prism (v. 9.0). Continuous data with a normal distribution were expressed as mean ± standard deviation (SD), while categorical variables were presented as counts or percentages. Prior to analysis, normal distribution was confirmed using P-P plots. To compare cardiac strain function parameters between the conventional bSSFP and three CS cine sequences, a paired *t*-test was utilized. The Wilcoxon signed-rank test was used to compare image contrast between every two cine sequences. The reproducibility of LV strain values was examined utilizing the intraclass correlation coefficient (ICC) for inter- and intra-observer consistency. The strain values of the LV derived from identical cine sequences (inter-observer = first and second measurements by YW, intra-observer = by YW and PCL) were compared. Statistical significance was set at a *p*-value of less than 0.001 (two-tailed).

## Result

### Demographic

A total of 102 patients, including 75 males and 27 females, with a mean age of 46.5 ± 17.1 (SD) years (age range: 14–86 years), were included in this study. All recruited participants met the diagnostic criteria for cine image quality. The etiologies of the participants represented in the CMR examination comprised hypertrophic cardiomyopathy (*n* = 24), arrhythmia (*n* = 23), ischemic cardiomyopathy (*n* = 17), dilated cardiomyopathy (*n* = 13), myocarditis (*n* = 11), hypertension (*n* = 5), amyloidosis (*n* = 3), takotsubo cardiomyopathy (*n* = 1), rheumatic heart disease (*n* = 2), arrhythmogenic right ventricular cardiomyopathy (*n* = 1), glycogen shortage disease (*n* = 1), and sarcoidosis (*n* = 1) (detailed information can be found in Table 2).

**Table 2:** Demographic variables of the population (*n* = 104).

Characteristics	Patients (*n* = 102)	Range
Age (years)	46.5 ± 17.1	14–86
Sex (Female/Male)	27/75	–
Height (cm)	166.6 ± 6.0	146–188
Weight (kg)	69.2 ± 16.9	42–129
BMI (kg/m^2^)	24.4 ± 4.5	17.3–41.2
Main cardiovascular-related etiology		
HCM	24	–
Arrhythmia	23	–
ICM	17	–
DCM	13	–
Myocarditis	11	–
Hypertension	5	–
Amyloidosis	3	–
Takotsubo cardiomyopathy	1	–
Rheumatic heart disease	2	–
ARVC	1	–
Glycogen storage disease	1	–
Sarcoidosis	1	–

BMI, body mass index; HCM, hypertrophic cardiomyopathy; ICM, ischemic cardiomyopathy; DCM, dilated cardiomyopathy; ARVC, arrhythmogenic right ventricular cardiomyopathy.

### CMR parameters

**Figure 3:**
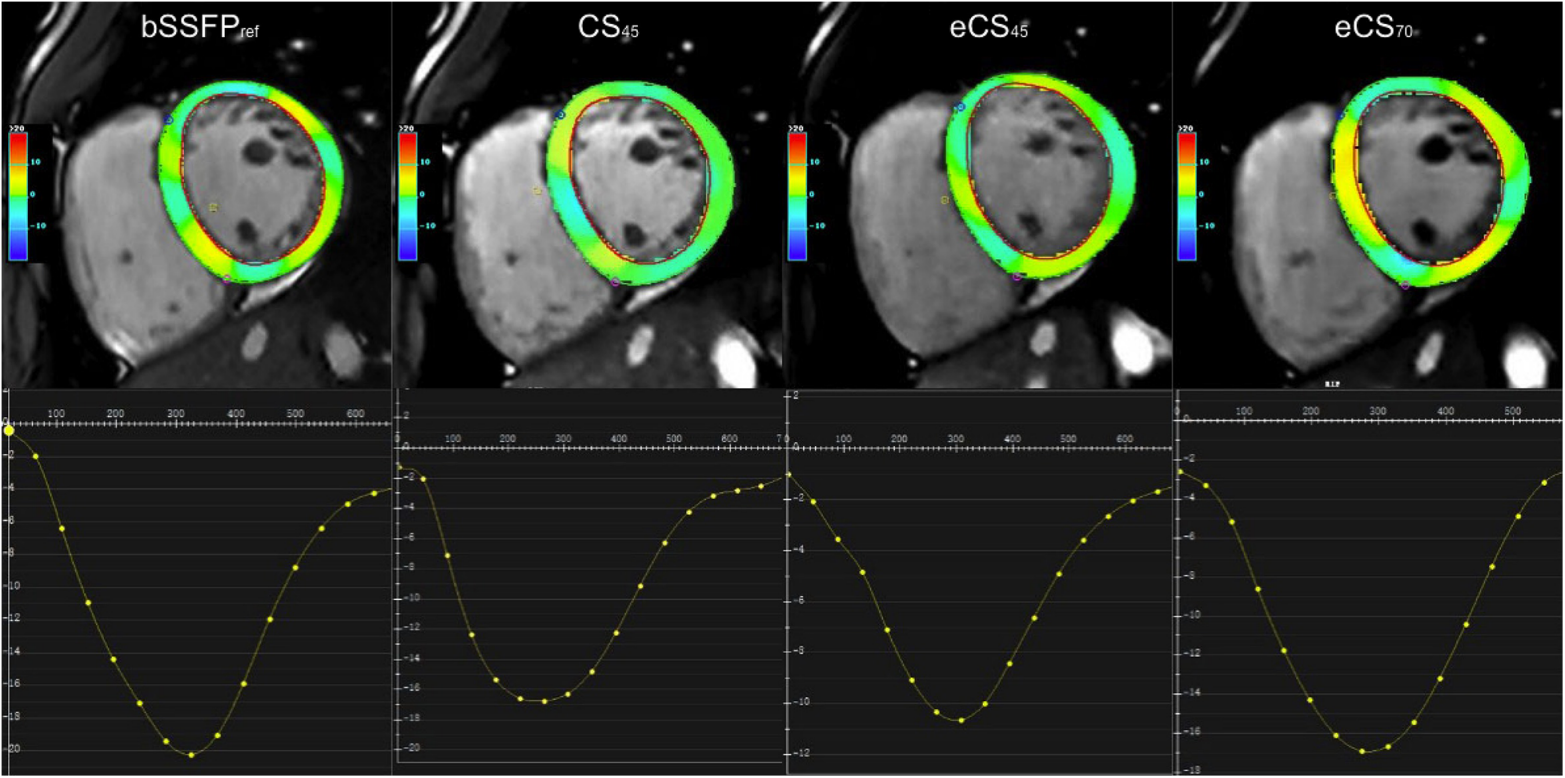
Strain analysis was performed using feature tracking on a stack of short-axis views. The global circumferential strain (GCS, %) graph of the left ventricular (slice 4) showed distinct results among the different cine sequences: referenced bSSFP_ref_ cine displayed the highest GCS peak value (−21.1), while eCS_45_ cine exhibited the lowest value (−10.5). CS_45_ and eCS_70_ cine sequences displayed almost identical GCS values. CS, compressed sensing; bSSFP, balanced steady-state free precession.

**Figure 4:**
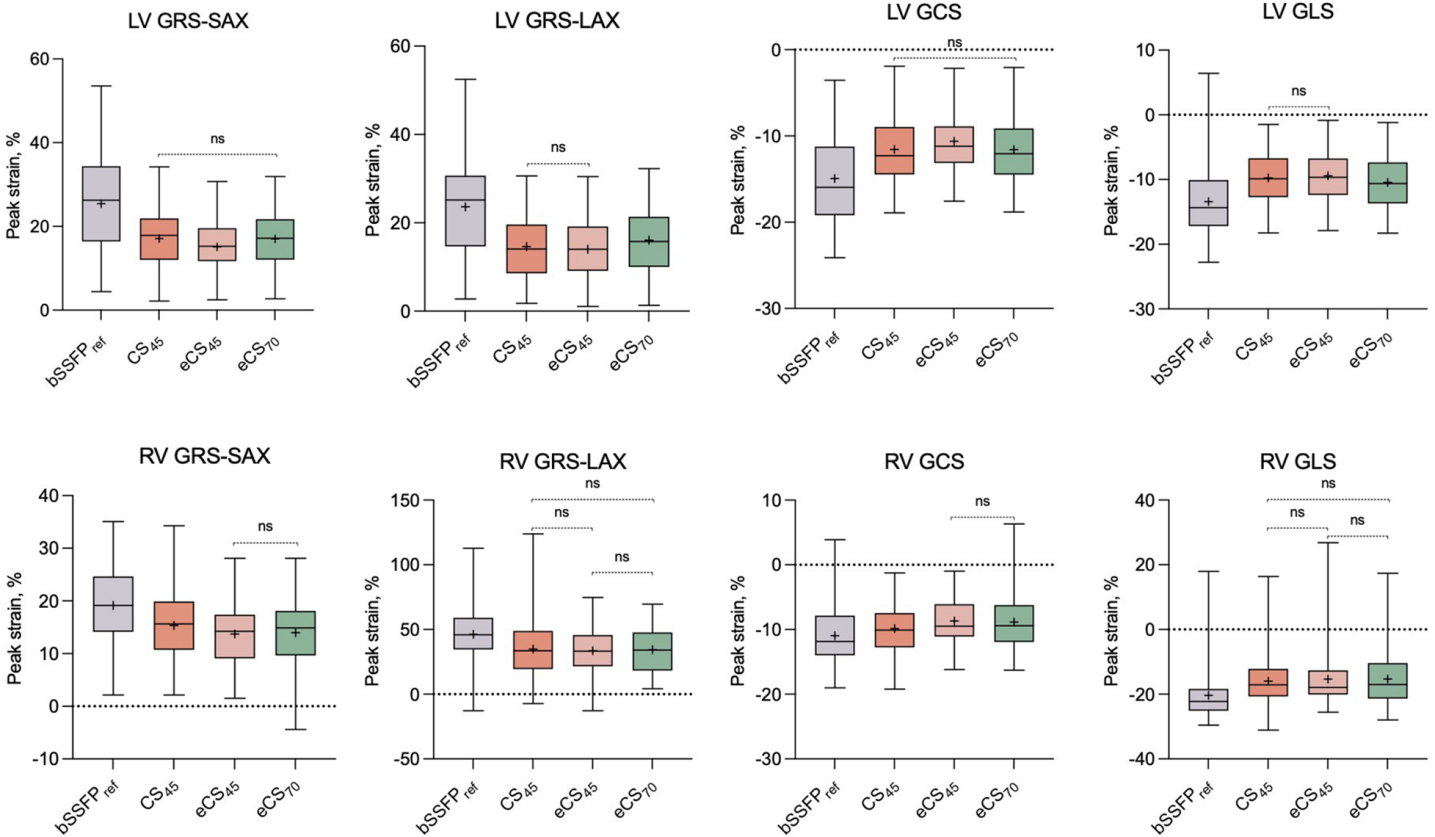
A set of box plots is used to summarize the strain results for the conventional bSSFP_ref_ cine and three CS cine (CS_45_, eCS_45_, eCS_70_) sequences. Non-significant comparisons between each pair of cine sequences are denoted as “ns”. LV, left ventricle; RV, right ventricle; GRS-SAX, global radial strain measured on short-axis slices; GRS-LAX, global radial strain measured on long-axis slices; GCS, global circumferential strain; GLS, global longitudinal strain; CS, compressed sensing; bSSFP, balanced free steady state precession. The symbol “+” denotes the mean value.

Compared to the routine bSSFP_ref_ cine, all three single breath-hold CS cine sequences yielded significantly lower global strain results, including biventricular GRS-LAX, GRS-SAX, GCS, and GLS (*p* < 0.001, example images shown in Figure 3 and Supplementary Figure S1). Following the administration of gadolinium contrast agent, there were no notable influences found on GRS-LAX and GLS values for the left and right ventricles, that derived from both enhanced and unenhanced CS cine sequences utilizing the same flip angle (CS_45_ and eCS_45_), meanwhile, significant reductions were observed in biventricular GRS-SAX and GCS values (*p* < 0.001). When increased flip angle of CS cine to 70 degrees after injection of gadolinium contrast agent (CS70), a significant elevation in LV strain values was noticed in comparison to CS45 (*p* < 0.001). This change had no noticeable impact on the strain parameters of the right ventricle. The increase in the flip angle of CS cine sequences appeared to compensate for the effect of the gadolinium contrast agent on LV GRS-SAX, LV GCS, RV GRS-LAX, and right ventricular (RV) GLS, except for LV GRS-LAX, LV GLS, RV GRS-LAX, and RV GLS (detailed information can be found in Table 3, Supplementary Table S1, and Figure 4). The inter- and intra-group consistency of LV strain results derived from bSSFP_ref_ and three CS cine sequences demonstrated a low variation and an outstanding agreement between identical and distinct observers. All ICC values exceeded 0.91. (all *p* < 0.001, detailed information can be found in Table 4 and Supplementary Table S2). Conventional bSSFP_ref_ and unenhanced CS cine sequences presented the same image contrast, meanwhile, the gadolinium agent decreased the image contrast of CS cine sequences regardless of the flip angle rising (see Table 3, *p* < 0.001).

**Table 3:** Comparison of biventricular strain parameters and image contrast across four different cine sequences.

	bSSFP_ref_^a^ (Mean^ ^± SD)	CS_45_^b^ (Mean^ ^± SD)	eCS_45_^c^ (Mean^ ^± SD)	eCS_7_^d^ (Mean^ ^± SD)	*P*-value					
					ab	ac	ad	bc	bd	cd
LV GRS-SAX	25.4 ± 11.4	17.1 ± 7.6	15.1 ± 6.2	17.0 ± 7.2	*	*	*	*	–	*
LV GRS-LAX	23.6 ± 10.8	14.6 ± 7.2	14.0 ± 6.6	16.1 ± 7.6	*	*	*	–	*	*
LV GCS	−14.9 ± 5.2	−11.6 ± 4.2	−10.6 ± 3.6	−11.6 ± 4.0	*	*	*	*	–	*
LV GLS	−13.4 ± 5.3	−9.7 ± 4.0	−9.4 ± 3.7	−10.4 ± 4.0	*	*	*	–	*	*
RV GRS-SAX	19.2 ± 8.0	15.3 ± 6.6	13.7 ± 6.1	14.0 ± 6.2	*	*	*	*	*	–
RV GRS-LAX	46.2 ± 19.4	34.9 ± 19.0	33.7 ± 16.8	34.5 ± 17.6	*	*	*	–	–	–
RV GCS	−11.0 ± 4.6	−9.9 ± 3.8	−8.7 ± 3.7	−8.9 ± 4.1	*	*	*	*	*	–
RV GLS	−20.4 ± 7.2	−16.0 ± 7.4	−15.4 ± 8.5	−15.3 ± 8.0	*	*	*	–	–	–
Image contrast	2.5 ± 0.3	2.5 ± 0.3	1.7 ± 0.2	2.1 ± 0.3	–	*	*	*	*	*

LV, left ventricle; RV, right ventricle; GRS-SAX, global radial strain measured on short-axis slices; GRS-LAX, global radial strain measured on long-axis slices; GCS, global circumferential strain; GLS, global longitudinal strain; CS, compressed sensing; bSSFP, balanced free steady state precession.

^a^ bSSFP_ref_.

^b^ CS45.

^c^ eCS_45_.

^d^ eCS_70_.

* *p* < 0.001.

**Table 4:** Intra and inter-observer variability testing of left ventricular strain using intraclass coefficient (ICC) for 20 randomly selected patients.

	Intra-observer variability				Inter-observer variability			
	bSSFP_ref_	CS_45_	eCS_45_	eCS_70_	bSSFP_ref_	CS_45_	eCS_45_	eCS_70_
GRS-SAX	0.984*	0.978*	0.974*	0.991*	0.995*	0.996*	0.990*	0.995*
GRS-LAX	0.944*	0.913*	0.943*	0.943*	0.995*	0.998*	0.987*	0.997*
GCS	0.988*	0.982*	0.975*	0.991*	0.996*	0.997*	0.990*	0.994*
GLS	0.942*	0.934*	0.964*	0.958*	0.993*	0.997*	0.990*	0.997*

GRS-SAX, global radial strain measured on the short-axis slice; GRS-LAX, global radial strain measured on the long-axis slice; GCS, global circumferential strain; GLS, global longitudinal strain; CS, compressed sensing; bSSFP, balanced free steady state precession.

* *p* < 0.001.

## Discussion

In recent years, significant research efforts have been devoted to accelerating CMR examination speed and reducing artifacts in CMR image examination. However, many previous studies have primarily concentrated on evaluating the feasibility and stability of ventricular function parameters in various heart diseases, comparing CS-based and conventional cine sequences, without fully exploring the potential of CMR-FT for strain assessment in clinical and research settings (20–22). In the realm of clinical practice, the widespread adoption of CS cine CMR holds great potential for patients with impaired cardiac and respiratory function. However, it is important to note that CS cine CMR workflows can vary significantly across different clinical centers (to reduction scanning time mostly, such as performing the cine sequence before the late gadolinium enhanced sequence). Moreover, the interpretation of CS cine-based CMR-FT, being a relatively new tool, requires more informed analysis. Limited research has been conducted to explore the impacts of compressive sensing cine techniques and scan parameters on myocardial strain values in patients with different CVDs. To address this gap, we have developed an examination protocol that includes four parallel cine sequences on both short and long-axis views. This protocol aims to assess the consistency of strain parameters obtained from bSSFP_ref_ cine and CS cine sequences, as well as to examine the influence of flip angle and gadolinium contrast agents on these parameters in patients with various types of heart diseases in clinical CMR examination.

The current study's key discovery reveals that all three CS cine sequences of 102 patients with cardiovascular diseases exhibit significantly decreased biventricular GRS-SAX, GRS-LAX, GCS, and GLS values in comparison to the conventional cine sequence. This observation suggests that the CS cine, used in our study as a single breath-hold high-speed technique, may not furnish equivalent strain values for patients with various cardiovascular conditions even with almost the same acquisition parameters (see Table 1). This discovery fails to replicate the strain value comparison results observed in other studies between CS-based cine and conventional cine, which demonstrate only minor differences (23–25). The accurate functioning of the FT algorithm is heavily reliant on achieving a high contrast between the blood and myocardium (26). Former studies have identified that the diminished blood-to-myocardium contrast resulting from highly accelerated compressed sensing acquisition can adversely impact the accurate delineation of myocardial contour, leading to the erroneous detection of partial myocardium (27). This might have provided a potential explanation for the discrepancies seen in the measurement of global strain values. However, in our study, the conventional bSSFP_ref_ and unenhanced CS cine sequences exhibited similar image contrast, rendering them unable to serve as a justification for our strain findings. CS cine imaging can mitigate respiratory movement, which may lead to k-space inconsistencies between different segments and result in increased artifacts and blurring of the MR image, potentially affecting the delineation of endocardial and epicardial contours by CVI^42^ or the observers (28). The short-axis CS cine sequence, which could be completed in only one breath-hold, differs from the conventional short-axis cine (9.1 ± 0.6 times). This difference in breath-hold can result in slice displacement between the two measurements, which in turn may affect the consistency of strain results. Moreover, notable differences in global myocardial strain measurements have been reported between commercially available software vendors. This is particularly true for the assessment of GLS and GRS, even though all of these software solutions are based on optical flow algorithms (18). The cine CS utilized in our study is based on the VALAS sampling pattern in k-space, and it was acquired from patients diagnosed with various heart ailments, further exploration is required to understand the effects of these conditions on the CVI^42^ CMR-FT analysis. We also observed that although both CS cine and bSSFPref cine were acquired over two heartbeats, there was significant variation in their systolic/diastolic phase. This discrepancy may also contribute to the inconsistency of strain values. Additionally, considering the differences in acquisition parameters (detailed in Table 1), it is probable that the lower temporal resolution of CS cine sequences may play a significant role in our findings. Previous studies have demonstrated that temporal resolution has a significant impact on strain and strain rate in CMR-FT deformation analyses (29). Specifically, the global radial strain (GRS) was found to decrease significantly when the number of cine cardiac phases was changed from 25 to 20 (30). Furthermore, when comparing left ventricular global longitudinal strain (GLS), GRS, global circumferential strain (GCS), and strain rate using breath-hold compressed sensing cine imaging at high and conventional temporal resolutions, it was reported that a higher temporal resolution resulted in noticeably greater cardiac strain measurements (31).

Contrast-enhanced cine images, while frequently exhibiting reduced blood-myocardium image contrast, demonstrated a notable decrease in both right and left ventricular GRS-SAX and GCS values within our study, consistent with previous research results conducted on conventional cine CMR-FT (27). In contrast, long-axis strain parameters, specifically GRS-LAX and GLS values, derived from CS cine sequences with the same flip angles, demonstrated a more resilient nature compared to other strain parameters. These long-axis strain parameters exhibited no significant variation before and after the injection of contrast agent. GLS is generally acknowledged as a highly robust and reproducible parameter for left ventricular deformation, and the utilization of biventricular GLS is recommended in CMR guidelines for quantitative assessment of both LV and RV function (32, 33). Furthermore, our observations revealed that an increase in the flip angle could mitigate the impact of the gadolinium contrast agent on various strain parameters obtained from CS cine sequences, similar to previous findings (34). Notably, this compensation effect was apparent in parameters such as GRS-LAX and GCS values of the left ventricle, as well as GRS-LAX and GLS values of the right ventricle. While previous studies have recommended a flip angle of 100 degrees, we opted for a lower value of 70 degrees (35). This decision was made to strike a balance between the initially low flip angle of 45 degrees and the potential risk of exceeding the specific absorption rate (SAR) limit associated with larger flip angles in obesity participants (27).

The study had several limitations that warrant consideration. Firstly, the data was gathered exclusively from a single clinical center, potentially introducing bias in participant demographics and clinical practices. Secondly, the data statistics process encompassed only more commonly used strain parameters, such as GCS, GLS, GRS-SAX, and GRS-LAX values of both left and right ventricles. Notably absent were other myocardial strain parameters, including strain rate and peak displacement, which diminishes the comprehensive applicability of our study. Thirdly, the study used different TE/TR and temporal resolution for CS Cine compared to bSSFP_ref_. Moving forward, it is imperative that future studies encompass a more diverse patient cohort and employ CS cine sequences optimized for temporal resolution and other MR parameters TR/TE to corroborate our principal findings and foster the widespread adoption of the CS technique across clinical CMR.

## Conclusion

Despite the introduction of gadolinium contrast agents and the utilization of larger flip angles, CS cine sequences consistently yielded diminished values for GRS, GCS, and GLS in both the left and right ventricles compared to their conventional bSSFP cine counterparts. Nevertheless, while the effect of contrast agents on left ventricular GRS and GCS can be mitigated by elevating the flip angle in CS cine sequences, such an adjustment had no impact on the GRS, GCS, and GLS values of the right ventricle. Consequently, further investigation is required to achieve optimal strain results, and the administration of single-breath hold CS cine should have its application conditions restricted.

## Data Availability

The original contributions presented in the study are included in the article/Supplementary Material, further inquiries can be directed to the corresponding author.

## Supplementary material

Supplementary Figure S1 Images of conventional bSSFP_ref_ cine and CS cine in two patients. Patient 1 was a 33-year-old man with paroxysmal arrhythmia. Patient 2 was a 52-year-old woman with ischemic cardiomyopathy. Conventional bSSFP_ref_ cine followed by CS cine images before and after contrast agent injection with a 45° flip angle, and contrast enhanced CS cine with a 70° flip angle were shown in panels A to D, and E to H. CS, compressed sensing; bSSFP, balanced free steady state precession.
