# Supplementary figures and images for: The effects of flip angle and gadolinium contrast agent on single breath-hold compressed sensing cardiac magnetic resonance cine for biventricular global strain assessment
Source: Front Cardiovasc Med. 2024 Jan 29;11:1286271. doi: 10.3389/fcvm.2024.1286271 (PMC10859435; doi:10.3389/fcvm.2024.1286271)

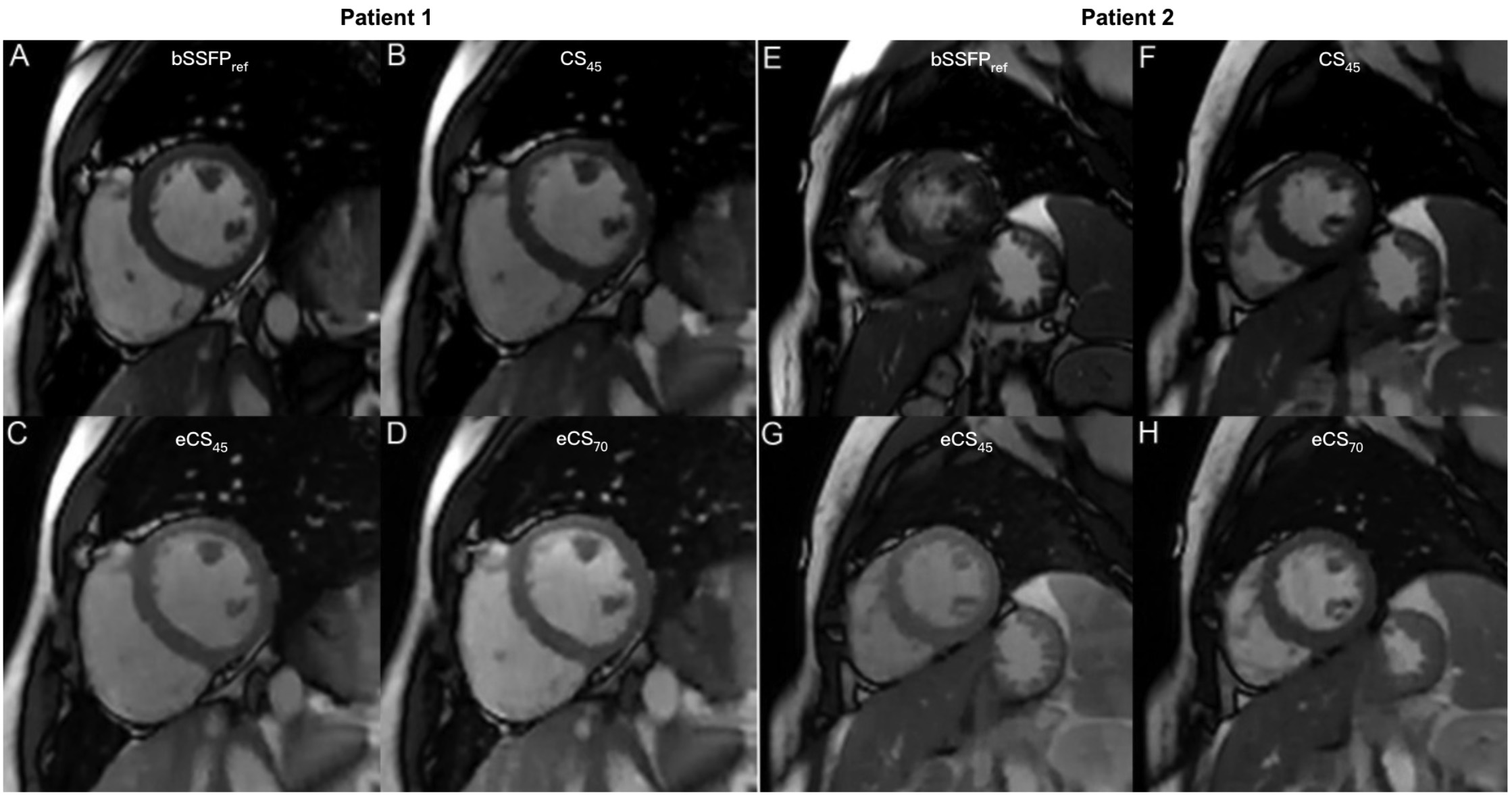

Supplement: Supplementary Figure S1 — Images of conventional bSSFPref cine and CS cine in two patients. Patient 1 was a 33-year-old man with paroxysmal arrhythmia. Patient 2 was a 52-year-old woman with ischemic cardiomyopathy. Conventional bSSFPref cine followed by CS cine images before and after contrast agent injection with a 45° flip angle, and contrast enhanced CS cine with a 70° flip angle were shown in panels A to D, and E to H. CS, compressed sensing; bSSFP, balanced free steady state precession. [file Image1.jpeg]
