# Supplementary material for: The effects of flip angle and gadolinium contrast agent on single breath-hold compressed sensing cardiac magnetic resonance cine for biventricular global strain assessment
Source: Front Cardiovasc Med. 2024 Jan 29;11:1286271. doi: 10.3389/fcvm.2024.1286271 (PMC10859435; doi:10.3389/fcvm.2024.1286271)
Supplement: Supplementary file 2 [file Table1.docx]

Table S1. Comparison of biventricular feature tracking parameters across four different cine sequences using paired t-test

|  | t value | | | | | | | | | | | |
| --- | --- | --- | --- | --- | --- | --- | --- | --- | --- | --- | --- | --- |
|  | ab  (t) | 95% CI | ac  (t) | 95% CI | ad  (t) | 95% CI | bc  (t) | 95% CI | bd  (t) | 95% CI | cd  (t) | 95% CI |
| LV GRS-SAX | **16.6** | 7.3-9.3 | **16.0** | 9.0-11.1 | **15.8** | 7.3-9.4 | **6.3** | 1.4-2.6 | 0.3 | -0.4-0.5 | **-7.3** | -2.4--1.4 |
| LV GRS-LAX | **15.2** | 7.8-10.1 | **14.9** | 8.3-10.9 | **13.4** | 6.4-8.6 | 1.8 | -0.1-1.3 | **-4.8** | -2.0--0.8 | **-5.6** | -2.8--1.3 |
| LV GCS | **-18.4** | -3.7-0.2 | **-17.6** | -3.8--4.8 | **-16.9** | -2.9--3.7 | **-6.0** | -0.6--1.3 | 0.3 | -0.2-0.3 | **7.5** | 0.7-1.3 |
| LV GLS | **-13.1** | -3.1--4.2 | **-14.4** | -3.4--4.6 | **-12.2** | -2.4--3.4 | -1.8 | -0.7-0.1 | **4.6** | 0.4-1.0 | **6.9** | 0.7-1.3 |
| RV GRS-SAX | **11.1** | 3.1-4.5 | **11.6** | 4.5-6.3 | **12.4** | 4.3-6.0 | **4.7** | 0.9-2.3 | **4.4** | 0.7-1.9 | -0.9 | -0.9-0.3 |
| RV GRS-LAX | **7.4** | 8.3-14.3 | **6.9** | 8.9-16.2 | **7.1** | 8.5-15.1 | 0.7 | -2.0-4.5 | 0.3 | -2.3-3.2 | -0.7 | -3.1-1.5 |
| RV GCS | **-5.2** | -1.5--0.6 | **-8.4** | -2.8--1.7 | **-7.4** | -2.7--1.5 | **-6.0** | -1.6--0.8 | **-4.1** | -1.5--0.5 | 0.6 | -0.3-0.6 |
| RV GLS | **-6.6** | -5.7--3.1 | **-5.6** | -6.7--3.2 | **-6.3** | -6.6--3.5 | -0.7 | -2.3-1.2 | -1.0 | -2.0-0.7 | -0.1 | -1.5-1.3 |

LV, left ventricle; RVEDV, right ventricle; RVESV, right ventricular end-systolic volume; GRS-SAX, global radial strain measured on short-axis slices; GRS-LAX, global radial strain measured on long-axis slices; GCS, global circumferential strain; GLS, global longitudinal strain; CS, compressed sensing; bSSFP, balanced free steady state precession. a = bSSFP_ref_，b = CS_45_, c = eCS_45_, d = eCS_70_, 95% CI = 95% confidence interval of the difference, t = t value.
