# Supplementary material for: The effects of flip angle and gadolinium contrast agent on single breath-hold compressed sensing cardiac magnetic resonance cine for biventricular global strain assessment
Source: Front Cardiovasc Med. 2024 Jan 29;11:1286271. doi: 10.3389/fcvm.2024.1286271 (PMC10859435; doi:10.3389/fcvm.2024.1286271)
Supplement: Supplementary file 3 [file Table2.docx]

Table S2.

Intra-observer variability of left ventricular strain with 95% CI

|  |  |  |  | Intra-observer variability | | | |  |
| --- | --- | --- | --- | --- | --- | --- | --- | --- |
|  | bSSFP_ref_ | 95% CI | CS_45_ | 95% CI | eCS_45_ | 95% CI | eCS_70_ | 95% CI |
| GRS-SAX | 0.984** | 0.962-0.994 | 0.978** | 0.940-0.992 | 0.974** | 0.936-0.990 | 0.991** | 0.976-0.997 |
| GRS-LAX | 0.944** | 0.862-0.977 | 0.913** | 0.794-0.964 | 0.943** | 0.865-0.977 | 0.943** | 0.864-0.977 |
| GCS | 0.988** | 0.971-0.984 | 0.982** | 0.953-0.993 | 0.975** | 0.934-0.990 | 0.991** | 0.976-0.996 |
| GLS | 0.942** | 0.820-0.979 | 0.934** | 0.841-0.973 | 0.964** | 0.913-0.986 | 0.958** | 0.898-0.983 |

GRS-SAX, global radial strain measured on the short-axis slice; GRS-LAX, global radial strain measured on the long-axis slice; GCS, global circumferential strain; GLS, global longitudinal strain; CS, compressed sensing; bSSFP, balanced free steady state precession. **=p <0.001.

Inter-observer variability of left ventricular strain with 95% CI

|  |  |  |  | Inter-observer variability | | | |  |
| --- | --- | --- | --- | --- | --- | --- | --- | --- |
|  | bSSFP_ref_ | 95% CI | CS_45_ | 95% CI | eCS_45_ | 95% CI | eCS_70_ | 95% CI |
| GRS-SAX | 0.995** | 0.988-0.998 | 0.996** | 0.991-0.999 | 0.990** | 0.975-0.996 | 0.995** | 0.988-0.998 |
| GRS-LAX | 0.995** | 0.988-0.998 | 0.998** | 0.994-0.999 | 0.987** | 0.967-0.995 | 0.997** | 0.991-0.999 |
| GCS | 0.996** | 0.989-0.998 | 0.997** | 0.993-0.999 | 0.990** | 0.975-0.996 | 0.994** | 0.986-0.998 |
| GLS | 0.993** | 0.883-0.997 | 0.997** | 0.994-0.999 | 0.990** | 0.974-0.996 | 0.997** | 0.993-0.999 |

GRS-SAX, global radial strain measured on the short-axis slice; GRS-LAX, global radial strain measured on the long-axis slice; GCS, global circumferential strain; GLS, global longitudinal strain; CS, compressed sensing; bSSFP, balanced free steady state precession. **=p <0.001.
